# A Randomized and Controlled Crossover Study Investigating the Improvement of Walking and Posture Functions in Chronic Stroke Patients Using HAL Exoskeleton – The HALESTRO Study (HAL-Exoskeleton STROke Study)

**DOI:** 10.3389/fnins.2019.00259

**Published:** 2019-03-29

**Authors:** Matthias Sczesny-Kaiser, Rebecca Trost, Mirko Aach, Thomas A. Schildhauer, Peter Schwenkreis, Martin Tegenthoff

**Affiliations:** ^1^Department of Neurology, BG University Hospital Bergmannsheil Bochum, Bochum, Germany; ^2^Department of Spinal Cord Injury, BG University Hospital Bergmannsheil Bochum, Bochum, Germany; ^3^Department of General and Trauma Surgery, BG University Hospital Bergmannsheil Bochum, Bochum, Germany

**Keywords:** stroke rehabilitation, exoskeleton, hybrid assistive limb, physiotherapeutic approach, locomotor training

## Abstract

**Background:** The exoskeleton HAL (hybrid assistive limb) has proven to improve walking functions in spinal cord injury and chronic stroke patients when using it for body-weight supported treadmill training (BWSTT). Compared with other robotic devices, it offers the possibility to initiate movements actively. Previous studies on stroke patients did not compare HAL-BWSTT with conventional physiotherapy (CPT). Therefore, we performed a crossover clinical trial comparing CPT and HAL-BWSTT in chronic stroke patients with hemiparesis, the HALESTRO study. Our hypothesis was that HAL-training would have greater effects on walking and posture functions compared to a mixed-approach CPT.

**Methods:** A total of 18 chronic stroke patients participated in this study. Treatment consisted of 30 CPT sessions and of 30 sessions of BWSTT with a double leg type HAL exoskeleton successively in a randomized, crossover study design. Primary outcome parameters were walking time and speed in 10-meter walk test (10MWT), time in timed-up-and-go test (TUG) and distance in 6-min walk test (6MWT). Secondary outcome parameters were the functional ambulatory categories (FAC) and the Berg-Balance Scale (BBS). Data were assessed at baseline, at crossover and at the end of the study, all without using and wearing HAL.

**Results:** Our study demonstrate neither a significant difference in walking parameters nor in functional and balance parameters. When HAL-BWSTT was applied to naïve patients, it led to an improvement in walking parameters and in balance abilities. Pooling all data, we could show a significant effect in 10MWT, 6MWT, FAC and BBS, both therapies sequentially applied over 12 weeks. Thereby, FAC improve from dependent to independent category (3 to 4). One patient dropped out of the study due to intensive fatigue after each training session.

**Conclusion:** HAL-BWSTT and mixed-approach CPT were effective therapies in chronic stroke patients. However, compared with CPT, HAL training with 30 sessions over 6 weeks was not more effective. The combination of both therapies led to an improvement of walking and balance functions. Robotic rehabilitation of walking disorders alone still lacks the proof of superiority in chronic stroke. Robotic treatment therapies and classical CPT rehabilitation concepts should be applied in an individualized therapy program.

## Introduction

Stroke is a growing medical and socioeconomical problem these days (Feigin et al., 2015). Epidemiologic studies estimated an yearly incidence of 800,000 in the United States to 1.0 million in the European Union (EU) (Truelsen et al., 2006; Mozaffarian et al., 2016). Incidence and prevalence increased over the last 20 years, while mortality decreased remarkably due to improving emergency medicine (Reeves et al., 2008; Koton et al., 2014). Stroke incidence is supposed to raise to 1.5 million per year in 2025 in the EU (Truelsen et al., 2006). Total costs of stroke care were expected to increase up to $184.1 billion in the United States for the year 2030 (Ovbiagele et al., 2013). In the next years, stroke therapy will become an even greater burden for national socioeconomic systems. While acute stroke therapies mainly focus on reducing infarcted brain tissue, reducing expected acute functional deficits and stroke survival, rehabilitation therapies in chronic stroke patients usually focus on restoration and reducing existing and persisting functional deficits. Both treatment approaches are necessary to lower resulting costs for the public healthcare system. Therefore, studies in both stroke settings (acute/chronic) are needed to limit persisting disabilities and analyze the best possible rehabilitation options.

Today, it is generally accepted that outpatient physiotherapy and other therapies would not lead to a significant functional recovery in chronic stroke.

The functional recovery curve reaches saturation after 6 months with only few fluctuations (Duncan et al., 1994; Jørgensen et al., 1995; Kwakkel et al., 2004; Langhorne et al., 2011). However, only few studies have addressed whether modern rehabilitation tools could induce significant functional recovery even in chronic stages. First positive evidence was given by innovative robotic devices for arm and walking training (Kwakkel et al., 2004; Huang and Krakauer, 2009; Reinkensmeyer et al., 2009; Lo et al., 2010). The results indicated that functional recovery might be possible even in chronic stages of stroke. The implementation of recent scientific knowledge on neurorehabilitation and neuronal plasticity like task-specificity, context-specificity and/or high-intensity and repetitive practice is a great advantage of new rehabilitation approaches. Several different robotic devices for locomotor support have been developed over the last 10 years. Most of them serve primarily as a medical and nursing device for walking support, but can be used as a training tool as well. For example, the ReWalk exoskeleton (ReWalk Robotics Ltd., Yokneam, Isreal) and the Indego bionic exoskeleton (Parker Hannifin Corporation, Cleveland, OH, United States) allow people with paraparesis due to spinal cord injury (SCI) to stand up, walk with a defined pattern and climb stairs. Induced locomotion is passive, not neurological self-induced and not based on any biological signal. Both robots use pre-programmed walking patterns that were executed irrespective of patient’s remaining walking abilities. ReWalk and Indego have the intention to be applied predominantly as a walking aid for outdoor use. For walking rehabilitation, different robotic devices and gait trainers have been developed (e.g., Locomat, Gait Trainer GT a. s. o.). Even though, scientists showed therapeutic effects on walking parameters and disability, so far, in larger studies, they failed to show superiority when compared with conventional physiotherapies (Mehrholz and Pohl, 2012; Chang and Kim, 2013; Swinnen et al., 2014). Neither Locomat nor Gait Trainer GT uses neurobiological signal for locomotion control.

In contrast, the exoskeleton hybrid assistive limb (HAL) is controlled voluntarily by the patient’s own muscle signals detected by surface electrodes. This self-initiated movement is capable to induce a somatosensory feedback-loop that enhances neural plasticity and locomotor learning (Sczesny-Kaiser et al., 2015). Pilot studies on patients with SCI, and chronic stroke showed safety and beneficial effects on walking functions (Kawamoto et al., 2013; Aach et al., 2014; Cruciger et al., 2014; Nilsson et al., 2014; Yoshimoto et al., 2015; Grasmücke et al., 2017; Jansen et al., 2017). In SCI, our study group demonstrated that HAL-assisted and body-weight supported treadmill training with supervision of a specialized physiotherapist led to a significant improvement of walking parameters and ambulatory capacity as indicated by the Walking Index for SCI II. These effects were observed in acute and chronic SCI patients, even up to 19 years after ictus (Aach et al., 2014; Grasmücke et al., 2017). Treadmill- and HAL-associated parameters like walking distance, speed and time as well as independent parameters like 10-meter walk test and 6-min walk test increased significantly up to 50% (Grasmücke et al., 2017). Moreover, these improvements could be detected in chronic tetraplegic and paraplegic patients. Older age (>50 years) and spastic motor behavior were non-significant negative predictors for walking endurance improvements. In stroke patients, similar results for HAL-assisted and body-weight supported treadmill training were demonstrated by several study groups in Japan and Sweden (Wada et al., 2010; Kawamoto et al., 2013, 2014; Nilsson et al., 2014; Yoshimoto et al., 2015) The therapeutic target was hemiparesis in all studies, and predominantly, patients with hemispheric insult were enrolled. In addition to technical requirements, safety and feasibility (Kawamoto et al., 2014; Nilsson et al., 2014), effects on treadmill-bound and treadmill-independent parameters were investigated (Kawamoto et al., 2013; Yoshimoto et al., 2015, 2016; Mizukami et al., 2017). Again, study results showed significant improvements, and even promising results indicating significant improvements in the functional ambulatory category (FAC) (Kawamoto et al., 2013). In spite of these encouraging positive results of HAL-training, most of these studies were performed in an uncontrolled design, e.g., using a conventional and mixed physiotherapy setup as control group. Thus, these results encourage to perform controlled studies using HAL-assisted treadmill training in chronic stroke patients. To become an established part of neurorehabilitation programs, HAL has to be compared with conventional physiotherapy (CPT) which is the cornerstone, today. Here, Bobath’s concept and proprioceptive neuromuscular facilitation (PNF) were used regularly (Dickstein et al., 1986; Lincoln et al., 1999; Luke et al., 2004; Wang et al., 2005).

We hypothesized that, in chronic stroke patients, HAL-assisted body-weight supported treadmill training would be more effective in recovery of walking parameters than CPT (provided according to current standards of practice). We further hypothesized that exoskeletal HAL training would improve outcome parameters reflecting functional independence more than conventional therapy.

## Materials and Methods

### Study Design and Patients

**FIGURE 1:**
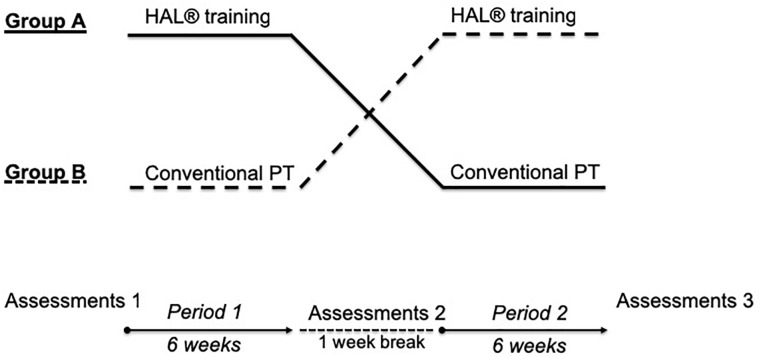
Study design. Two-period, controlled crossover design. Each period included 30 therapy sessions. 1-week break was held between both periods. Before, at crossover and at the end of the study, assessments were done. HAL = hybrid assistive limb, Group 1 = HAL-CPT; Group 2 = CPT-HAL.

This study was performed in a monocentric, controlled, randomized, two-period crossover design to test the efficacy of HAL-assisted body-weight supported treadmill training compared to CPT on walking parameters in chronic stroke patients. The study was done in accordance with the Declaration of Helsinki. It was approved by the local Ethics Committee of the Medical Faculty of Ruhr University Bochum (reg. no. 4894-14). All patients provided written informed consent before participating. The study has been registered in German Clinical Trials Register (DRKS-ID: DRKS00006821). Figure 1 shows our study design. Patients were randomly assigned to Group 1 or to Group 2 using a computer-generated list. Masking to treatment allocation for therapist and patients was not feasible. 18 ambulatory, chronic stroke patients with incomplete hemiparesis were enrolled. The inclusion criteria were (1) incomplete paresis of the leg after single incident of ischemic or hemorrhagic stroke, at least 6 months prior and (2) age between 18 and 75 years. Exclusion criteria were (1) severely impaired communication due to native language or aphasia, (2) impaired cardiorespiratory capacity, (3) severe multimodal neglect, (4) history of severe infection or history of infection with multiresistant bacteria, (5) complete paralysis of the leg, (6) history of more than 1 stroke, (7) decubital ulcer of the lower extremities and the sacral region, (8) severe osteoporosis and fractures, (9) history of deep vein thrombosis and pulmonary embolism, (10) contracture of the leg, (11) body weight > 100 kg, (12) epilepsy, (13) electric medical devices like cardiac pacemaker, (14) metal implants like ventriculoperitoneal shunt. To characterize patients’ impairments in activities of daily living at baseline and cognition, the Barthel index, the FAC and the Montreal Cognitive Assessment (MoCA) were assessed (Holden et al., 1986; Nasreddine et al., 2005; Hachinski et al., 2006; Sivakumar et al., 2014). All demographic and clinical characteristics of the patients are shown in Tables 1, 2. Except patient no. 7 (pontine infarction), all patients had hemispheric lesions supplied by the middle cerebral artery.

**Table 1:** Demographic and clinical characteristics of the patients.

#	Sex	Age	Group	Time since stroke, months	Etiology	Side of paralysis	Assistive device	FAC	Barthel index	MoCA
1	m	52	1	108	ischemia	L	walking cane	4	100	22
2	m	65	1	81	ischemia	L	walking cane	4	100	21
3	m	61	1	69	hemorrhage	R	walking cane	5	100	21
4	f	61	1	95	ischemia	R	wheelchair	2	70	22
5	m	73	1	81	ischemia	L	walking cane	3	90	16
6	f	70	2	355	ischemia	R	none	4	100	17
7	m	62	2	10	ischemia	L	walking cane	4	100	18
8	f	57	1	30	hemorrhage	R	wheeled walker	4	100	28
9	m	72	2	32	ischemia	L	walking cane	4	100	25
10	m	71	1	45	ischemia	L	wheelchair	2	85	21
11	m	60	2	115	ischemia	L	wheelchair	4	80	23
12	m	69	2	204	ischemia	R	FES^∗^	5	100	23
13	m	71	1	24	hemorrhage	R	walking cane	5	100	26
14	m	57	2	26	hemorrhage	R	none	5	100	23
15	m	58	2	120	ischemia	L	none	5	95	28
16	f	74	2	27	ischemia	L	none	4	85	21
17	f	58	1	29	ischemia	L	none	4	80	26
18	m	75	2	30	ischemia	R	wheelchair	0	45	20

*f = female, m = male, L = left, R = right, FAC = functional ambulation categories. Patient #17 was a drop-out because of massive fatigue after each training session. Group 1 = HAL-CPT, Group 2 = CPT-HAL; ^∗^FES = functional electrical stimulation for drop foot (ActiGate^®^).*

**Table 2:** Demographic and clinical characteristics of the groups.

	Group 1	Group 2	*p*-value
*n*	9	9	
Age (mean)	63	66	0.371
Sex (m/f)	6/3	7/2	NA
Time since stroke, months (mean)	62	102	0.331
Side of paralysis (L/R)	5/4	5/4	NA
Type of stroke (ischemic/hemorrhage)	6/3	8/1	NA
FAC (mean)	3.6	3.9	0.730
Barthel index (mean)	92	89	0.760
MoCA (mean)	22.6	22.0	0.740

*f = female, m = male, L = left, R = right, FAC = functional ambulation categories, MoCA = Montreal Cognitive Assessment. Group 1 = HAL-CPT; Group 2 = CPT-HAL; NA = not assessable.*

### HAL Therapy

**FIGURE 2:**
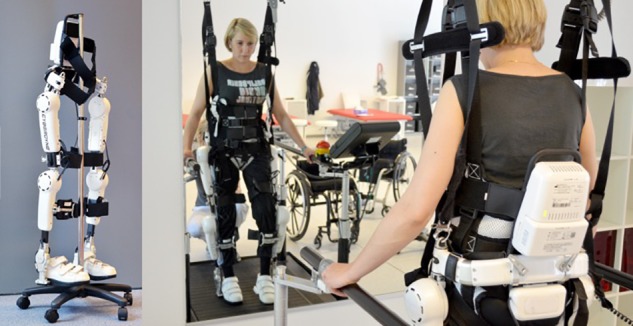
HAL exoskeleton and training setting. Left part of the figure shows HAL exoskeleton with its electronic actuators for hip and knee joints (“power units”), its battery pack and controller at the top and its EMG-electrodes and cables for detection of bioelectrical signals. Right part of the figure demonstrates the training setting with the treadmill, the body-weight support and the exoskeleton. Official picture of Cyberdyne Inc., the person was not subject of the published study and has been engaged and paid for commercials. Copyright Cyberdyne, Inc., published with kindly permission.

In both Groups, all patients underwent a 6-week HAL-assisted, supervised, body-weight supported treadmill training (BWSTT). Each patient was scheduled for a 30 min training session 5 times a week, resulting in 30 sessions. The therapy was supervised by one to two stroke-specialized and HAL-qualified physiotherapists. HAL is an exoskeleton with a patient size-adjustable frame and robotic actuators that attaches to the patient’s lower limbs. The joint movements are supported by electric motors. The exoskeleton percutaneously detects minimal bioelectric signals initiated by the patient’s voluntary muscle activities (hip and knee flexors and extensors) via electromyography (EMG) electrodes and/or the floor reaction force signals caused by patient’s intended weight shifts. Through a cable connection between the exoskeleton and the patient, this system allows a voluntary robotic-supported range of motion (cybernic voluntary control mode = CVC mode) to occur at each motor and joint separately. The electric motor support at the hip and knee joints is gradually adjustable according to the patient’s own remnant flexor and extensor muscle function. This leads to independent individual hip and knee joint motion support synchronous with the patients’ voluntary drive, so it enables individualized and adjustable muscle group locomotion training to be established for bilateral hip and knee flexors and extensors. The CVC mode has been used for the paretic leg. For the non-paretic leg, the CIC mode (cybernic impedance control) was chosen. It compensates for the weight of the device and the friction, without having any therapeutic effect. With the treadmill system (Woodway USA, Inc., Waukesha, WI, United States), the walking speed could be adjusted from 0 km/h to approximately 4.5 km/h. During treatment, the velocity of the treadmill was set individually between comfortable and maximum speed tolerated by the patient. Initially, the harness system supported approximately 25 to 50% of each patient’s body weight. This was individually reduced in subsequent training sessions depending on patient’s feedback and therapeutic progress. HAL robot suit and training setting are presented in Figure 2.

### Conventional Physiotherapy

All patients received CPT 5 times a week for 6 weeks resulting in 30 sessions. Patient #10 missed two sessions because of logistic trouble resulting in 28 session. All other patients underwent 30 HAL-sessions. The therapy was performed by one stroke-specialized physiotherapist. Each session lasted about 30–45 min individually based on patient’s daily condition. CPT was performed as mixed intervention consisting of different approaches and concepts, e.g., Bobath’s neurophysiological concept, PNF and motor (re-)learning programs referenced to Carr and Shepherd (1987). Main therapeutic aims were gait stability, physiological walking pattern, utilization of assistive devices, controlled activation of distinct muscle groups, and regulation of spasticity.

### Primary Outcomes: Walking Performance

The 10-meter walking test (10MWT) was measured at each training session. It detects the time to walk a 10 m distance (Bohannon, 1997; Perera et al., 2006). The timed-up-and-go test (TUG) describes the time and assistance required for standing up from a chair, to walk 3 meters, turn around, walk back and sit down (Podsiadlo and Richardson, 1991). The 6-min walking test (6MWT) evaluates the distance covered over a time of 6 min (Harada et al., 1999). The TUG test and 6MWT were assessed at baseline, at crossover and at the end of the study. All tests were performed without HAL exoskeleton.

### Secondary Outcomes: Functional and Balance Performance

The FAC is a functional walking test to evaluate ambulation ability. Patients were rated on 5 categories (0 = patient cannot walk, 5 = patient can walk independently anywhere) (Holden et al., 1986; Mehrholz et al., 2007). We used FAC for classification of our patients group as well as a secondary outcome parameter. Previous studies using HAL in subacute stroke patients reported a significant improvement in FAC score (Nilsson et al., 2014; Watanabe et al., 2014). The Berg-Balance Scale (BBS) is an assessment tool to evaluate balance skills and to predict falls. It contains of 14 items with a 5-point (range from 0 to 4) scale. Maximum score is 56 points. Static and dynamic activities of varying difficulty have to be performed. A score of <45 points indicates individuals with a higher risk of falls (Berg et al., 1992; Flansbjer et al., 2012). Both measurements have been tested at baseline, at crossover and at the end of the study.

### Statistics

At first, to rule out carry-over effects and test our study for validity a pre-test was performed by calculating the sum of the measured values in the periods for each patient and compared across the two groups by an unpaired *t*-test (Wellek and Blettner, 2012). When *p* was greater than 0.05, we proved that no carryover effects existed and that our “wash-out” phase was sufficiently long enough (1 week). As a second step, we calculated the within-subject difference in our outcome parameters between both study periods. To prove a statistical difference between the treatment effects HAL and CPT, two-sided Student’s unpaired *t*-test was carried out. Significance was assumed at the 5% alpha level (*p* > 0.05). If no statistical effect for one therapy was present, repeated measurement analysis of variance (rmANOVA) was performed to assess significant differences between both Groups (1 = HAL-CPT vs. 2 = CPT-HAL). We defined “time” as within-subject factor and “Group” as between-subject factor. Where it was appropriate, *post hoc t*-test were subsequently applied. For these tests, the significance level was adjusted by dividing it by the Number of comparisons (0.05/3 = 0.017; Bonferroni correction). Additionally, for pure analysis of HAL-BWSTT effects on walking parameters, BBS and FAC within therapy-naïve patients, Student’s two-sided, paired *t*-test has been performed with data of the first therapy period. This analysis was not the primary aim of the HALESTRO study, but it has been important to evaluate the therapeutic effect of HAL itself and, therefore, to enable a comparison of our data with previous work from other HAL-groups.

## Results

### Demographic and Clinical Characteristics of Both Groups

We did not find statistical differences for “age” and “time since stroke” between both groups (age: mean Group 1 = 63 years, mean Group 2 = 66 years, *p* = 0.371; time since stroke: mean Group 1 = 62 months, mean Group 2 = 102 months, *p* = 0.331). Looking at clinical characteristics like independence in activities of daily living (Barthel index), ambulation ability (FAC) and cognitive impairment (MoCA), statistical analysis revealed no significant differences between Group 1 and 2 (Barthel index: mean Group 1: 92, mean Group 2: 89, *p* = 0.760; FAC: mean Group 1 = 3.6, mean Group 2 = 3.9, *p* = 0.730; MoCA: mean Group 1: 22.6, mean Group 2: 22.0, *p* = 0.740). For results see Table 2.

### Crossover Analysis

**FIGURE 3:**
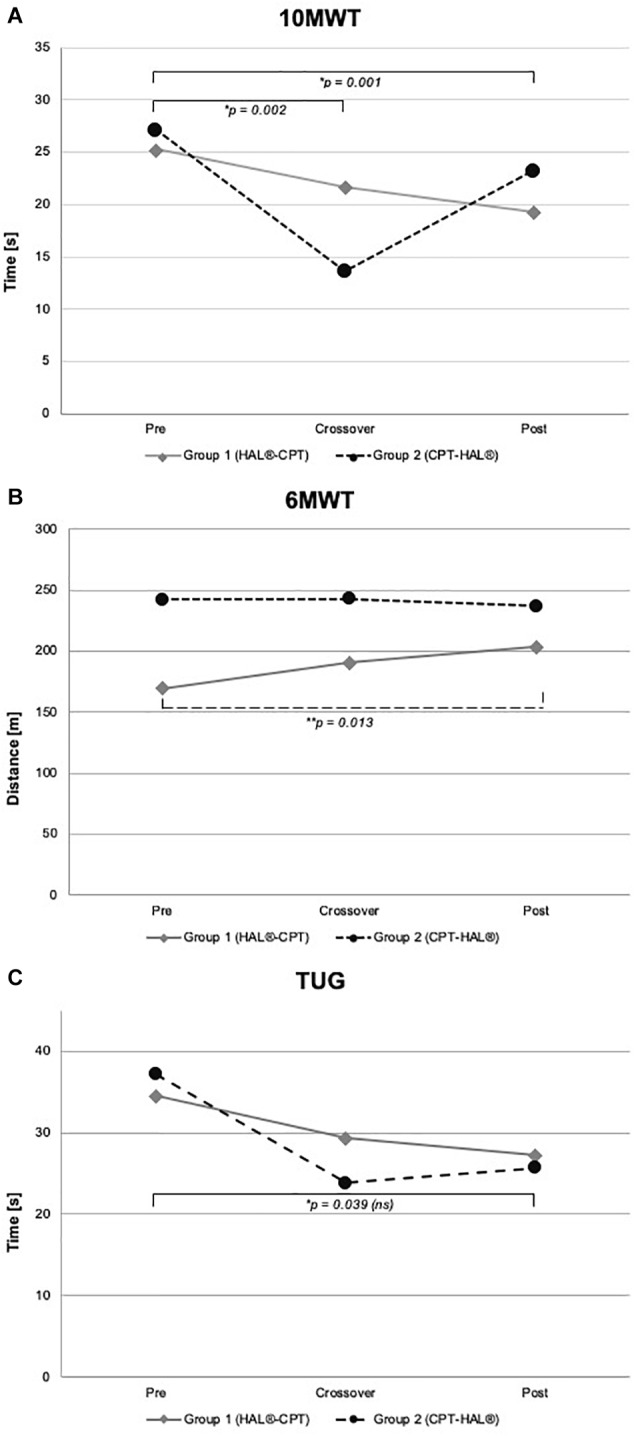
Walking performance parameter. Walking abilities at baseline, crossover and at the end of the study. Figure shows mean values for Group 1 (HAL-CPT) and Group 2 (CPT-HAL). Ten MWT = 10-meter walking test **(A)**, 6 MWT = 6-min walking test **(B)**, TUG = timed-up-and-go test **(C)**. ^∗^ indicated *post hoc t*-tests after significant effect for factor “time,” ^∗∗^ indicated *post hoc t*-test after significant interaction between “time” and “Group” (see “Results” section for details). *p*-threshold < 0.017 (Bonferroni correction).

The first step of our analysis was to look for carryover effects between both study periods. Statistical analysis revealed no carryover effects for all primary outcome parameters (walking abilities: 10MWT: *p* = 0.805, 6MWT: *p* = 0.529 and TUG: *p* = 0.692) as well as for the secondary outcome parameters FAC and BBS (FAC: *p* = 0.244, BBS: *p* = 0.949). As the next step, we looked for significant differences between the therapeutic effects of “HAL-BWSTT” and “CPT.” None of primary and secondary outcome parameters showed a significant difference between both treatments (10MWT: *p* = 0.071, 6MWT: *p* = 0.840, TUG: *p* = 0.835, FAC: *p* = 0.088, BBS: *p* = 0.737). Table 3 shows mean values for walking parameters, standard deviation and mean period effects. Figure 3, 4 demonstrate results of our primary and secondary outcome parameters. Additionally, whisker-box plots and scatter plots are available in the Supplementary Materials.

**Table 3:** Data sheet with mean values for walking parameters, standard deviation and mean period effects.

10MWT [s] and *speed (m/s)*						
	**Pre**		**Crossover**		**Post**	
	***mean***	***SD***	***mean***	***SD***	***mean***	***SD***
Group 1 (HAL^®^-CPT)	25.29	13.66	21.72	11.46	19.34	8.99
Group 2 (CPT-HAL^®^)	27.15	35.25	13.68	4.57	23.28	30.23
Group 1 (HAL^®^-CPT)	0.49	0.21	0.56	0.23	0.60	0.22
Group 2 (CPT-HAL^®^)	0.64	0.29	0.80	0.26	0.73	0.3

**Training effect 10MWT [s], mean of intraindividual differences**						

Group 1 (HAL^®^-CPT)	–2.69			±2.21		
Group 2 (CPT-HAL^®^)	–3.77			±7.42		

**6MWT [m]**						

	**Pre**		**Crossover**		**Post**	
	***mean***	***SD***	***mean***	***SD***	***mean***	***SD***

Group 1 (HAL^®^-CPT)	169.33	81.87	190.38	87.98	203.25	86.53
Group 2 (CPT-HAL^®^)	242.50	132.15	243.06	102.62	236.78	115.03

**Training effect 6MWT [m], mean of intraindividual differences**						

Group 1 (HAL^®^-CPT)	+12.88			±22.61		
Group 2 (CPT-HAL^®^)	+15.83			±34.66		

**TUG [s]**						

	**Pre**		**Crossover**		**Post**	
	***mean***	***SD***	***mean***	***SD***	***mean***	***SD***

Group 1 (HAL^®^-CPT)	34.54	23.79	29.32	17.27	27.22	20.26
Group 2 (CPT-HAL^®^)	37.20	55.56	23.83	5.42	25.65	33.86

**Training effect TUG [s], mean of intraindividual differences**						

Group 1 (HAL^®^-CPT)	+2.10			±10.61		
Group 2 (CPT-HAL^®^)	–1.46			±46.18		

*Ten MWT = 10-meter walk test, 6 MWT = 6-min walk test, TUG = timed-up-and-go, SD = standard deviation.*

**FIGURE 4:**
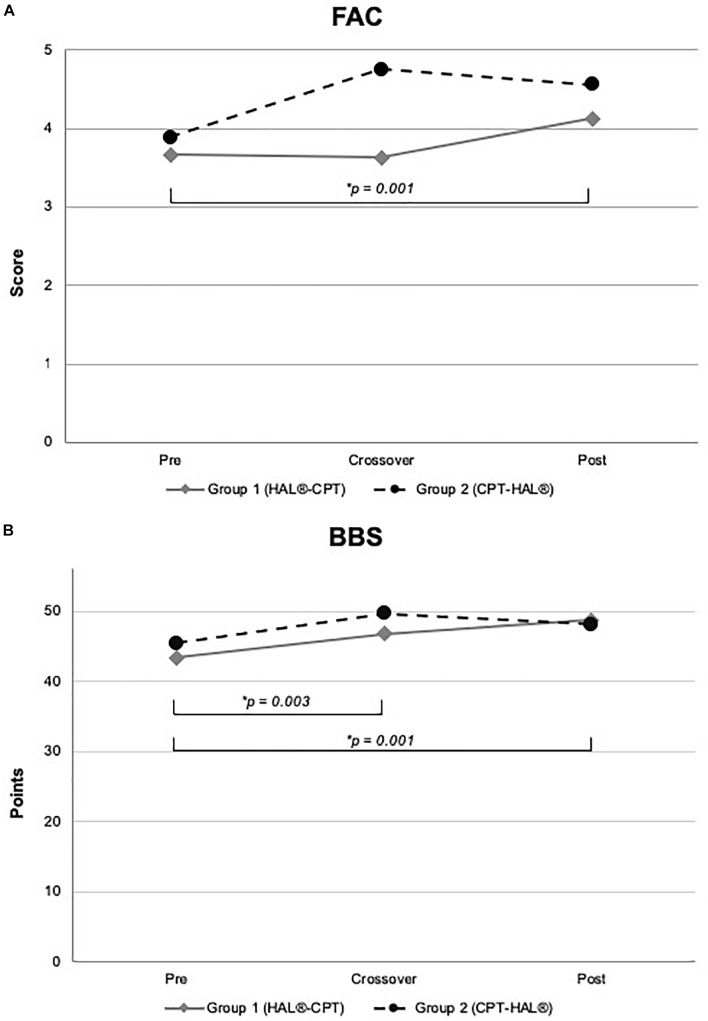
Functional outcome and Berg-Balance Scale. Figure shows mean values for Group 1 (HAL-CPT) and Group 2 (CPT-HAL). FAC = Functional ambulation categories **(A)**, BBS = Berg-Balance-Scale **(B)**. ^∗^ indicated *post hoc t*-tests after significant effect for factor “time.” *p*-threshold < 0.017 (Bonferroni correction).

### Analysis of Variance and *post hoc t*-Tests

We performed rmANOVA to show differences between both groups. For 10MWT, rmANOVA revealed a significant effect for the within-subject factor “time” (*F*_2,30_ = 14.459, *p* < 0.000). No significant interactions could be demonstrated for the interaction between “time” and between-subject factor “Group” (*F*_2,30_ = 1.346, *p* = 0.276) and for the between-subject factor “Group” (*F*_1,15_ = 0.028, *p* = 0.870). *Post hoc t*-test showed significant effects between baseline and crossover (*p* = 0.002), and between baseline and the end of the study (*p* = 0.001). For 6MWT, rmANOVA showed a significant interaction between the factors “time” and “Group” (*F*_2,30_ = 4.338, *p* = 0.022). All other rmANOVA analysis were without significant effects (within-subject factor “time”: *F*_2,30_ = 2.035, *p* = 0.148; between-subject factor “Group”: *F*_1,15_ = 0.819, *p* = 0.380). *Post hoc t*-test revealed significant effect for Group 1 (HAL-CPT) between baseline and end of the study (*p* = 0.013). Looking at the third primary outcome parameter TUG, statistical analysis demonstrated a significant effect for the within-subject factor “time” (*F*_2,30_ = 3.832, *p* = 0.033), but not for the interaction between “time” and “Group” (*F*_2,30_ = 0.248, *p* = 0.782). Again, between-subject factor “Group” revealed no significant effect (*F*_1,15_ = 0.020, *p* = 0.888). *Post hoc t*-tests revealed no significant differences (pre-cross: *p* = 0.06, cross-post: *p* = 0.993, pre-post: *p* = 0.039). For our secondary outcome parameter, significant effects were seen for the BBS. RmANOVA showed significance for the factor “time” (*F*_1,2_ = 12.294, *p* < 0.000). Again, interaction between “time” and “Group” and the between-subject factor “Group” itself revealed no significant effects (*F*_2,30_ = 1.801, *p* = 0.183; *F*_1,15_ = 0.015, *p* = 0.904). *Post hoc t*-tests showed significant effects between baseline and crossover assessments as well as between baseline and the end of the study (*p* = 0.003; *p* = 0.001). FAC data analysis showed significant effect for within-subject factor “time” (*F*_2,30_ = 9.900, *p* < 0.000) but not for interaction between “time” and “Group” (*F*_2,30_ = 2.363, *p* = 0.111) and the between-subject factor “Group” itself (*F*_1,15_ = 0.814, *p* = 0.381). *Post hoc t*-test showed significant effects between baseline measurements and the end of the study (*p* = 0.001).

### Analysis of First Period Effects in Group 1 (HAL-BWSTT Effects on Naïve Chronic Stroke Patients)

Student’s paired *t*-test showed significant effects of HAL-therapy on therapy-naïve patients for the following parameters: 10MWT (*p* = 0.003), 10MWT-speed (*p* = 0.001), TUG (*p* = 0.047) and BBS (*p* = 0.014). Mean values and standard deviations are presented in Table 3. No significant effect could be seen for 6MWT and FAC (*p* > 0.05).

### Analysis of First Period Effects in Group 2 (CPT Effects on Naïve Chronic Stroke Patients)

Student’s paired *t*-test showed no significant effects of mixed CPT on therapy-naïve patients for following parameters: 10 MWT (*p* = 0.099), TUG (*p* = 0.203), 6MWT (*p* = 0.197) and BBS (*p* = 0.081). A tendency can be observed for FAC (*p* = 0.051). Mean values and standard deviations are presented in Table 3.

## Discussion

The main finding of our study is that HAL-assisted BWSTT in chronic stroke patients did not improve walking functions and balance abilities significantly more than CPT in a mixed concept, each provided for 5 times a week for 6 weeks. Whereas, the sequential combination of both therapies, notwithstanding the order, achieved a significant improvement of walking functions, FAC and balance abilities. Although our study might stand partly in contradiction to other studies using HAL exoskeleton in stroke patients, it is the first HAL-study provided in a controlled, randomized and crossover design in chronic stroke. Besides, our study showed again that HAL-training itself can improve walking and balance functions in chronic stroke patients, like it has been reported by other study groups before (Kawamoto et al., 2013; Yoshimoto et al., 2016). The applied treatments have to be analyzed in detail to answer the question why robotic-assisted locomotor training with HAL failed to be more effective compared to CPT in chronic stroke patients.

### HAL-Treatment

We observed that our HAL-BWSTT protocol led to similar results as Kawamoto et al. (2013) and Yoshimoto et al. (2015) reported in their studies on chronic stroke patients. In our study, when HAL-BWSTT was applied to “naïve” patients (i.e., Group 1, first study period), it led to an improvement in walking parameters (10MWT, TUG) and in balance abilities (BBS), but not in functional ambulation. Comparing the mean values mentioned in the other studies with our data, our results are in line with previously published studies. Walking parameter in our study and in Kawamoto’s study improved by approximately 20%. Interestingly, in Yoshimoto’s study, the effect was greater, approximately 50%. Moreover, Yoshimoto and coworkers proved that HAL-training was more effective than CPT; their study was performed in a parallel group design. Although HAL-training was an effective therapy in our patients as well, there were several differences in the design of robotic training as well as in the CPT period. In our opinion, both issues might have contributed to different results at the end of our controlled, crossover study. First, Kawamoto et al. (2013) and Yoshimoto et al. (2015) did not apply (body-weight supported) treadmill training to their patients. Patients wore a harness connected to a mobile suspension system called the All-In-One Walking Trainer (Ropox A/S, Denmark) and walked freely on a floor with therapeutic assistance. Second, in both studies, the total number of HAL sessions and the frequency were considerably lower [Kawamoto et al. (2013): 16 sessions, 2/week, Yoshimoto et al. (2015): 8 sessions, 1/week, HALESTRO: 30 sessions, 5/week]. Third, and this might be the substantial difference to the HAL training in our study, Yoshimoto et al. (2015) have used a higher walking speed in order to compensate lower amount of training sessions. In contrast, we increased the number of sessions while using a lower walking speed. Both approaches are generally accepted concepts of modern stroke rehabilitation (Langhorne et al., 2011). Looking at our treadmill data (not published), Group 1 reached a median speed of 0.78 km/h (SD: ±0.22) to 1.34 km/h (SD: ±0.37) after 6 weeks of HAL training; after 6 weeks of CPT, Group 2 accomplished a median speed of 1.11 km/h (SD: ±0.28) to 1.48 km/h (SD: ±0.36) after 6 weeks of HAL-training. Yoshimoto et al. (2015) did not mention the individual walking speed on the treadmill. Less relevant, Yoshimoto et al. (2015) used the single leg version of the HAL robot suit (Kawamoto et al., 2009). Recently, it has been discussed how far single leg type is more suitable for patients with medium to low functional impairment and the double leg type is recommended for patients with severe functional impairment (indicated by FAC ≤ 1) (Nishimura et al., 2018). Studies investigating the efficiency of both types have not been performed yet. General recommendations do not exist. Therefore, we decided to apply the combination of CVC and CIC mode as mentioned above. Patients did not report discomfort or any disturbances. Summarizing, the HALESTRO study proved the efficiency of HAL robot suit for locomotor und posture functions using a high frequency BWSTT protocol with a comfortable treadmill speed. Like in classical walking training with a physiotherapist, HAL locomotor training offers the possibility of variations that are capable to modify the training outcome, like Yoshimoto’s study has shown. It remains unclear which training type is superior and which HAL type (single vs. double leg version) is recommended for which patient. Low-frequency and high-speed HAL training seems to be more effective.

### CPT in HALESTRO Study

Our study demonstrates no significant difference in walking, functional, and balance metrics between HAL-BWSTT and CPT. Because HAL-BWSTT has proven a significant effect after 6 weeks in naïve chronic stroke patients, one has to consider what was the characteristic of CPT in HALESTRO study. For the study design, it was very important for us to create a physiotherapeutic setting that is “real” and not artificially assembled only for the HALESTRO study. For this purpose, our physiotherapist was encouraged to perform an ambulatory care therapy that not only aimed on walking and gait but included the whole patient as a complex interrelated biological system. This might have been one reason why HAL-BWSTT effects were equalized. Again, we have to compare our results with Yoshimoto’s stroke study (Yoshimoto et al., 2016), especially because they could prove that HAL-therapy was significantly more effective than CPT. But, there were differences to our CPT design, leading to different results. At first, and the most important and most powerful difference was that we applied a so called “mixed” physiotherapy concept. In Pollock’s comprehensive meta-analysis, a number of CPT-approaches failed to prove superiority in rehabilitation of walking impairment after stroke (Pollock et al., 2007). Interestingly, it was insufficient to conclude that any therapy approach is more effective in promoting recovery of disability than any other. For physiotherapeutic intervention, using a “mix” of components from different approaches was more effective compared to a treatment control (Pollock et al., 2007). Indeed, it remained unclear which concept the therapists in Yoshimoto et al. (2015) have used. Instead of performing CPT one to 2 times a week for 40 min, absolutely no change of mean values of 10MWT, TUG and BBS were observed. Again, this is in contrast to our data; we clearly observed effects of CPT on all parameters. Since, there are basic differences in CPT approaches, our study failed to prove superiority of one therapeutic regime. CPT in our study was more effective than in Yoshimoto’s work. In fact, CPT is a strong instrument in walking rehabilitation as previous neurorehabilitation studies have demonstrated. Compared with the Locomat (Husemann et al., 2007; Mayr et al., 2007), with the Gait Trainer (Peurala et al., 2005), and with solely additional use of a treadmill (Duncan et al., 2011; Mehrholz et al., 2017a), hands-on physiotherapy based on “mixed” approach has proven its superiority and high efficiency on functional independence, walking speed and motor function (Pollock et al., 2007; Langhorne et al., 2009, 2011).

### Global Study Effects

Indeed, the HALESTRO study could not verify a significant superiority of robotic-assisted treadmill therapy with HAL or for hands-on physiotherapy for locomotor rehabilitation in chronic stroke patients. But our data have shown a clearly significant effect on the walking parameters 10MWT, 6MWT, FAC and BBS (see Figure 3, 4) when both therapies were sequentially applied over 12 weeks. For BBS and 10MWT, we have seen significant effects after the first study period (baseline – crossover). These significant effects were seen after pooling data of all subjects irrespective of therapeutic group and intervention/interventional sequence. An interesting detail is the improvement in FAC (see Table 4 and Figure 4). Although the functional recovery curve after stroke reaches saturation after 6 months with only few fluctuations, in our small study group of 18 resp. 17 patients, we could see a functionally important improvement depicted by FAC. The mean FAC exceeded the category level 3 and reached category level 4. Category 3 represents the ambulator-dependent level, category 4 the ambulator-independent level. So, patients could not only improve their results in the 10MWT or 6MWT, but also their daily relevant independency. As well as for FAC, patients benefit from enhanced balance functions and posture. Many patients reported a close relationship between both improvements; more stability led to more confidence, which led to more independency. However, these effects were not specific to one of both interventions, but for the combination of both therapies. This is a conclusion that not only we could state for our study, but also were made in greater and larger stroke rehabilitation studies. Recently, Mehrholz et al. (2017b) published an update of their Cochrane review examining the effects of electromechanical and robot-assisted gait training devices for improving walking after stroke very detailly. Finally, 36 trials with 1.472 participants were included. Outcome parameters were independency in walking, walking velocity and capacity also. Like in our study, the authors found in this meta-analysis that the combination of electromechanical-assisted training with physiotherapy after stroke is more likely to achieve independent walking than people who received gait trainings without those devices. Interestingly, the subgroup analysis of Mehrholz’ meta-analysis reported a result regarding HAL-therapy in stroke patients which can be confirmed by us: acute and subacute stroke patients as well as patients who are not ambulatory are more likely to achieve independent walking when they receive electromechanical-assisted gait training than without. Watanabe and coworkers have shown significant improvements in independent walking after 12 session of HAL therapy compared to conventional gait therapy (outcome parameter FAC). Their study has been performed in a parallel group design on 24 subacute stroke patients. Even though it was no primary aim of the HALESTRO study and a subgroup analysis would not be reliable due to small number of subjects, we also observed that the lower the FAC score and the lower the walking speed in 10MWT, the more improvement our patients could gain. It might be a ceiling effect. But Mehrholz et al. (2017b) results encourage to propose that there is a systematic effect behind this observation. In fact, our study does not allow any conclusions about this issue; further studies aiming on this topic should be carried out.

**Table 4:** Data sheet with mean values for functional ambulatory categories and Berg-Balance-Scale.

FAC						
	**Pre**		**Crossover**		**Post**	
	***mean***	***SD***	***Mean***	***SD***	***mean***	***SD***
Group 1 (HAL^®^-CPT)	3.67	1.12	3.63	1.19	4.13	1.13
Group 2 (CPT-HAL^®^)	3.89	1.54	4.75	0.46	4.56	1.01

**Training effect FAC, mean of intraindividual differences**					

Group 1 (HAL^®^-CPT)	0.5			± 0.53		
Group 2 (CPT-HAL^®^)	0.11			± 0.33		

**BBS [points]**						

	**Pre**		**Crossover**		**Post**	
	***mean***	***SD***	***mean***	***SD***	***mean***	***SD***

Group 1 (HAL^®^-CPT)	43.33	9.18	46.75	9.79	48.75	6.65
Group 2 (CPT-HAL^®^)	45.44	13.14	49.63	8.23	48.11	10.34

**Training effect BBS [points], mean of intraindividual differences**						

Group 1 (HAL^®^-CPT)	2.00			± 5.29		
Group 2 (CPT-HAL^®^)	1.33			± 2.40		

*FAC = functional ambulatory categories, BBS = Berg-Balance-Scale, SD = standard deviation.*

### Limitations

Certain limitations of our study should be noted. One point is its crossover design. For a better comparability of different therapeutic effects within one patient (each patient serves as her/his own control) and to avoid problems of comparability of study and control group, we decided to perform our study in a crossover design. To rule out a carryover effect, we specified a so called “washout phase” of 1 week. The duration of 1 week was assessed by other rehabilitation study previously performed in the field of stroke rehabilitation. Statistical analysis showed that no carryover effect was present. However, is it really possible to “washout” motor movements and coordinative motoric that one has learned within 1 week? Can these complex locomotor pattern really be forgotten within 1 week? Studies focusing extinction motor learning are ongoing. First evidence for distinct brain areas like the primary motor cortex could be delivered as areas of memory retrieval and extinction whereas the sensory cortices are supposed to be essential for long-term memory (Guo et al., 2017). A specific time period allowing for safe extinction or “washout” has not been identified so far. Statistically, we could deny any carryover effect for sure, but one must assume that certain new acquired locomotor sequences endured the “washout phase.” Therefore, these reflections underline the concept of synergistic effects of “conservative” physiotherapy and “innovative” robotic therapy. Moreover, it underlines the meaningfulness of combined therapy in crossover studies and not in parallel group design. Another limitation is the statistical power was low due to the small number of patients. One may note that the recruitment was very difficult. Furthermore, we missed to investigate the after effects. Notably, Watanabe and coworkers assessed follow-up examinations for subacute stroke patients and found promising results for HAL-intervention (Watanabe et al., 2017). Similar outlasting results have been reported by our group for SCI patients (Grasmücke et al., 2017; Jansen et al., 2017).

## Conclusion

In conclusion, our results indicate that BWSTT with HAL^®^ exoskeleton applied with moderate walking speed 5 times a week for 6 weeks (30 sessions) is not more effective in chronic stroke patients with moderate to severe impairment than mixed approach CPT. But both therapies combined sequentially, notwithstanding the order, is likely to achieve independent walking even in chronic stroke patients. The duration of these improvements remains unclear and further studies are needed.

## Data Availability

All datasets generated for this study are included in the manuscript and/or the Supplementary Files.

## Author Contributions

MS-K, RT, PS, and MT acquired and analyzed the data, participated in its design, and drafted the manuscript. MA and TS participated in the design and data collection and helped to draft the manuscript.

## Conflict of Interest Statement

TS was a consultant for Cyberdyne, Inc. at the time the study was conducted. The remaining authors declare that the research was conducted in the absence of any commercial or financial relationships that could be construed as a potential conflict of interest.
