# Supplementary figures and images for: A Randomized and Controlled Crossover Study Investigating the Improvement of Walking and Posture Functions in Chronic Stroke Patients Using HAL Exoskeleton – The HALESTRO Study (HAL-Exoskeleton STROke Study)
Source: Front Neurosci. 2019 Mar 29;13:259. doi: 10.3389/fnins.2019.00259 (PMC6450263; doi:10.3389/fnins.2019.00259)

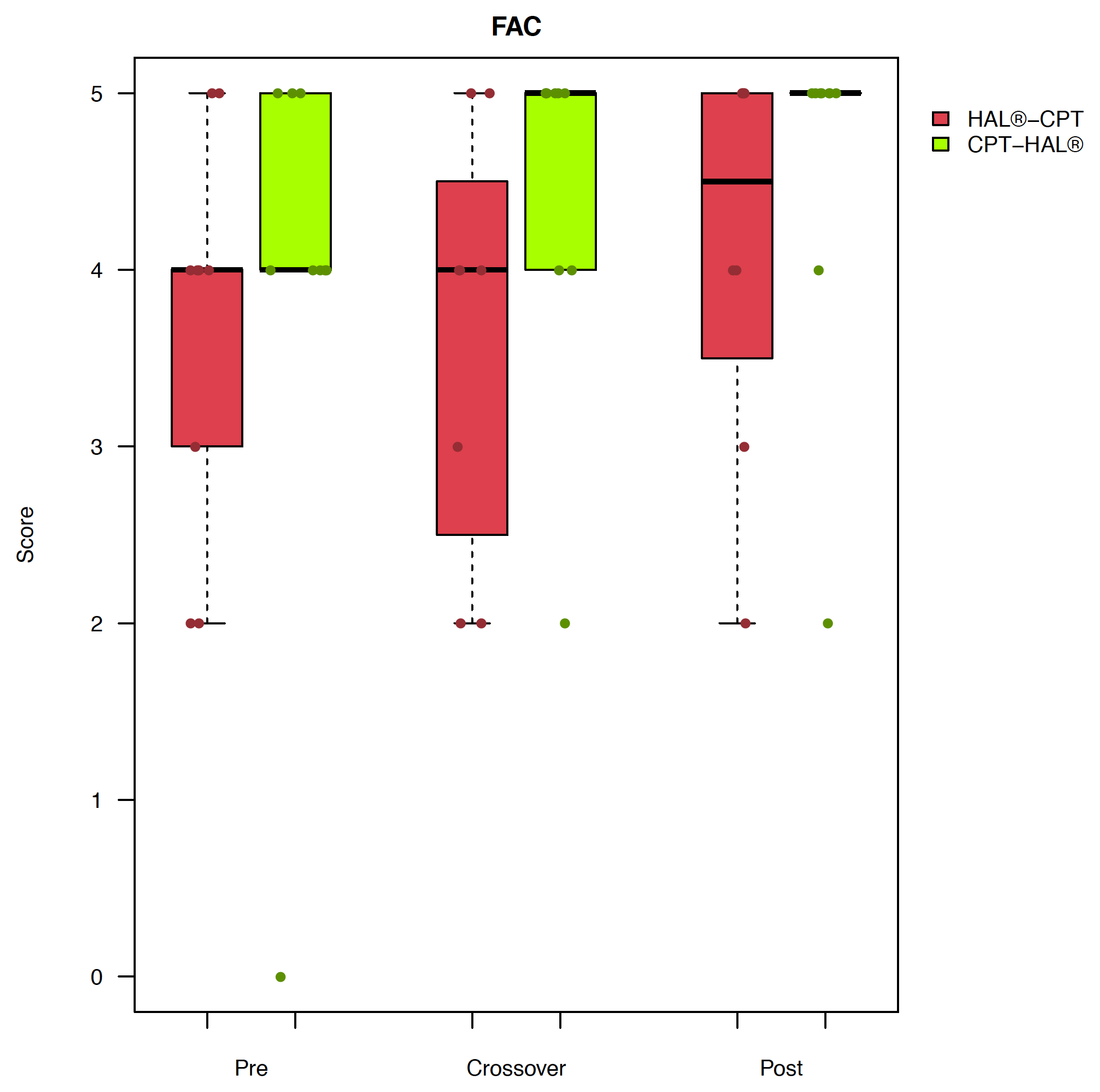

Supplement: Supplementary file 1 [file Image_1.TIF]

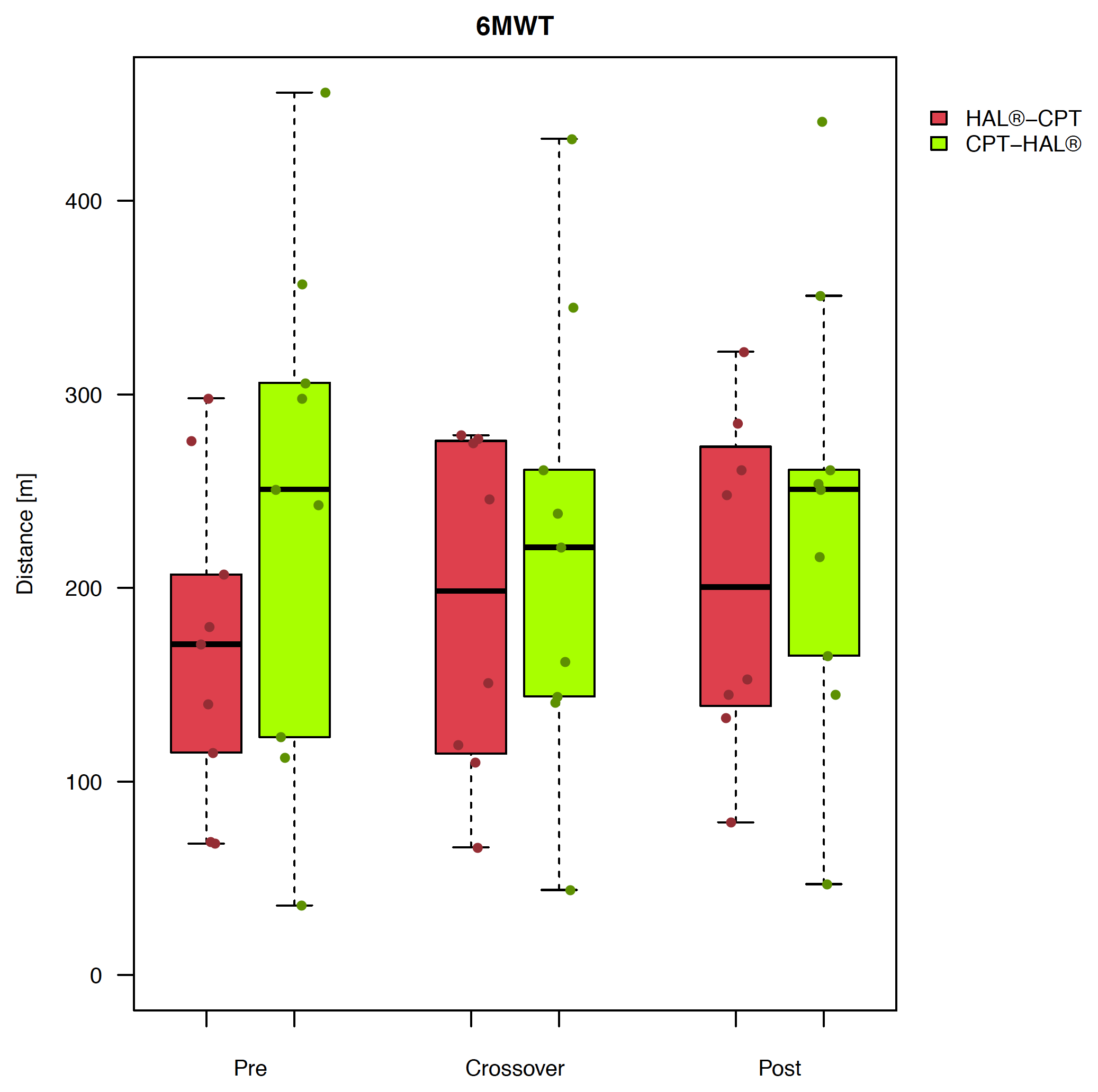

Supplement: Supplementary file 2 [file Image_2.TIF]

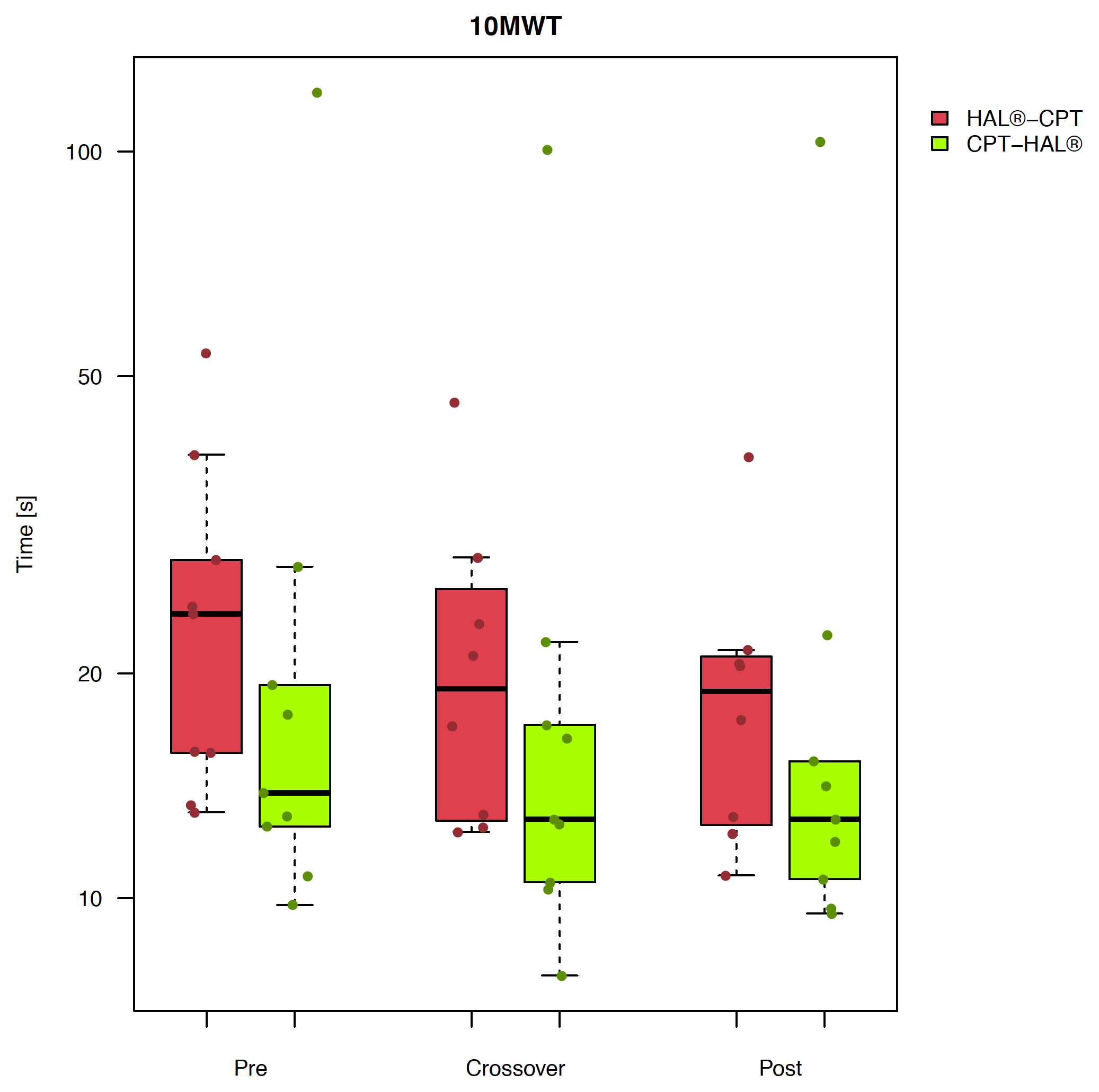

Supplement: Supplementary file 3 [file Image_3.TIF]

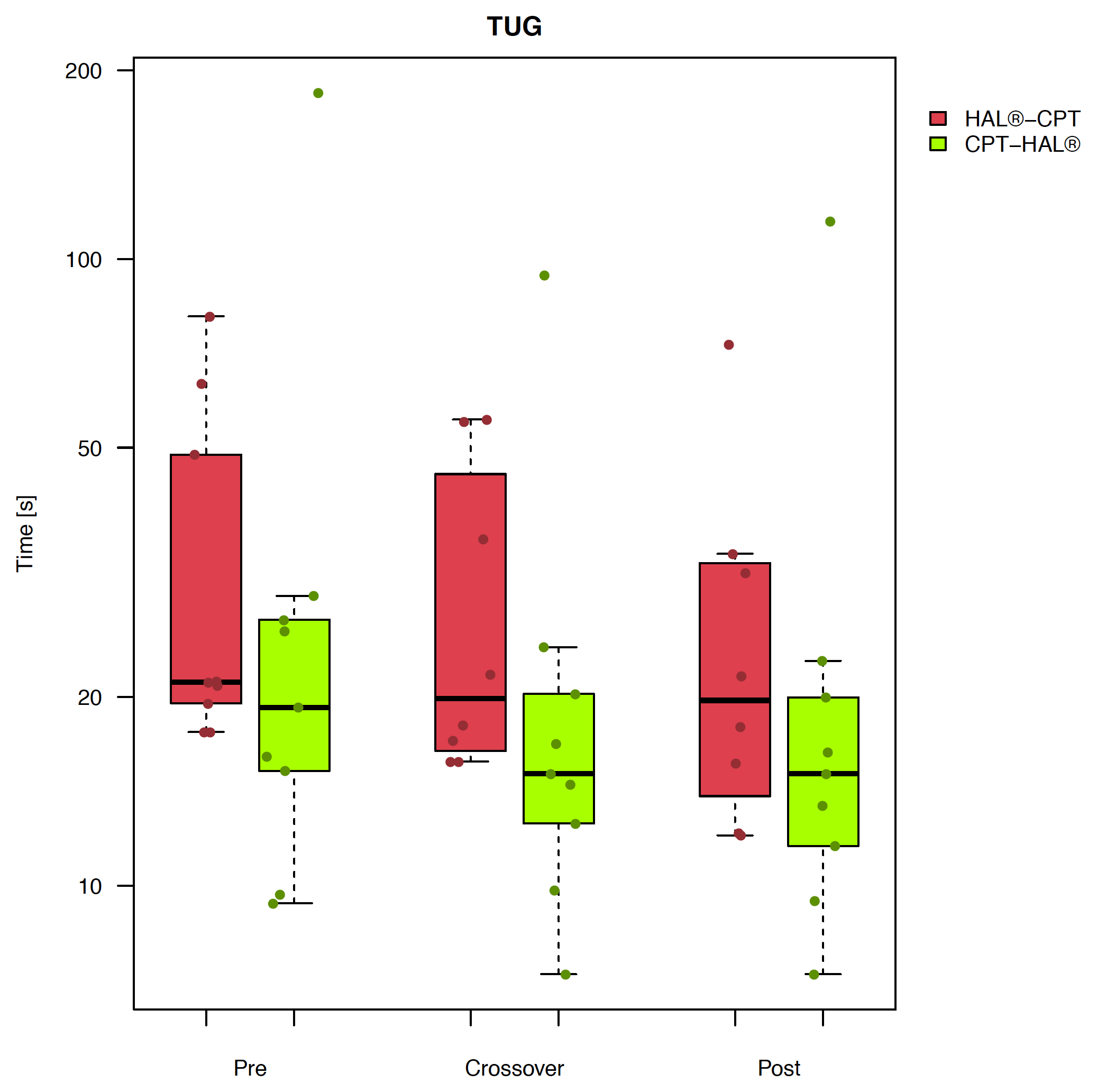

Supplement: Supplementary file 4 [file Image_4.TIF]

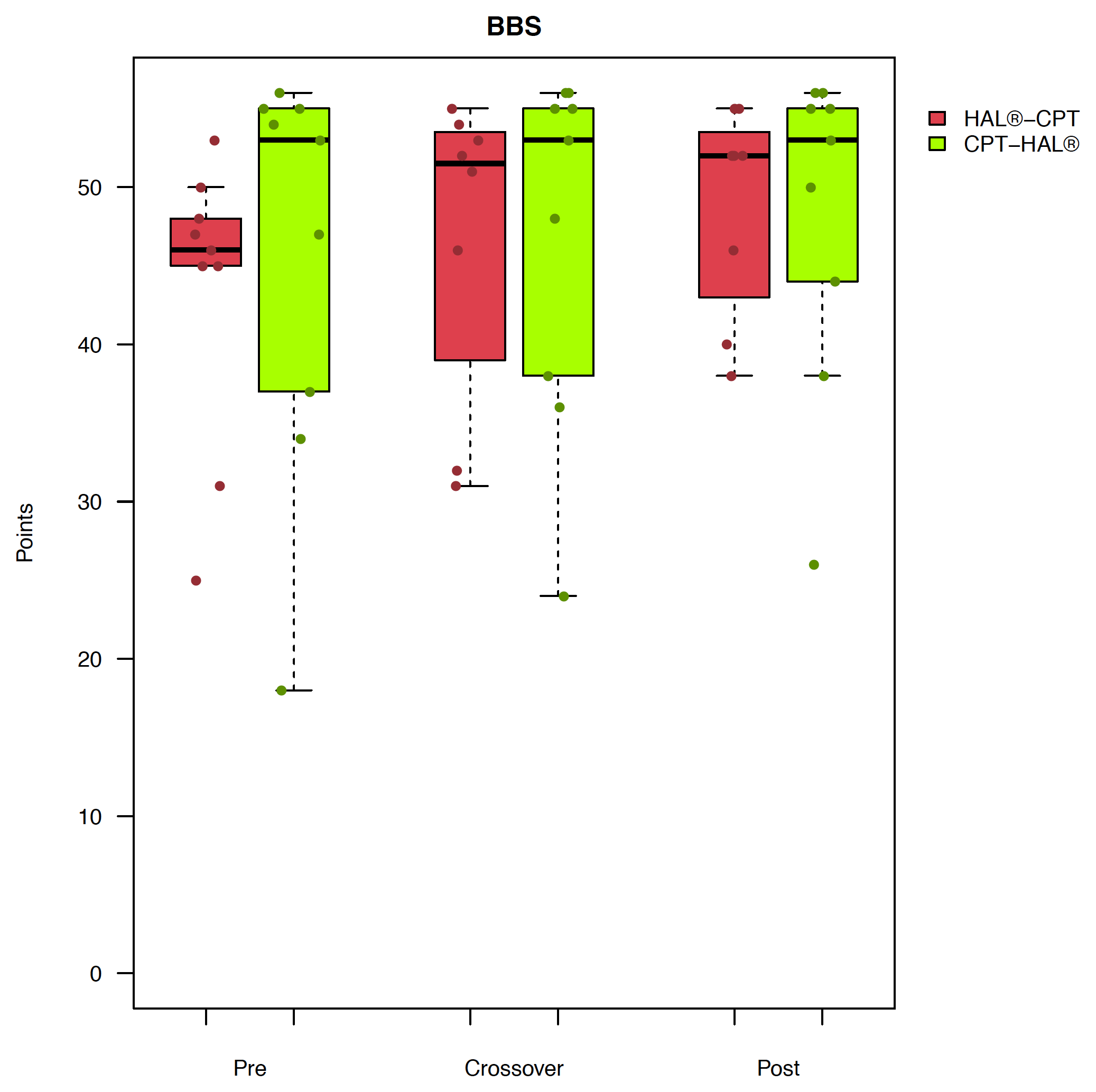

Supplement: Supplementary file 5 [file Image_5.TIF]
